# Integrative Digital Tools to Strengthen Data Management for Antimicrobial Resistance Surveillance in the “One Health” Domain in Nepal

**DOI:** 10.3390/tropicalmed8060291

**Published:** 2023-05-25

**Authors:** Santosh Kumar Yadav, Lilee Shrestha, Jyoti Acharya, Tulsi Ram Gompo, Sharmila Chapagain, Runa Jha

**Affiliations:** 1 National Public Health Laboratory, Kathmandu 44600, Nepal; 2 Central Veterinary Laboratory, Kathmandu 44600, Nepal

**Keywords:** AMR surveillance, data management, data standardization, digital tools, Nepal, One Health

## Abstract

Antimicrobial resistance (AMR) is increasing and represents one of the greatest public health challenges of our time, accounting for considerable morbidity and mortality globally. A “One Health” surveillance strategy, which integrates data concerning the resistant organisms circulating in humans, animals, and the environment, is required to monitor this issue and enable effective interventions. The timely collection, processing, analysis, and reporting of AMR surveillance data are necessary for the effective delivery of the information generated from such surveillance. Nepal has greatly improved its surveillance activities through a network of human and animal health laboratories; however, the data reported by sentinel laboratories are often inconsistent, incomplete, and delayed, causing challenges in terms of data cleaning, standardization, and visualization on a national level. To overcome these issues, innovative methods and procedures have been adopted in Nepal, with the development and customization of digital tools that reduce the human time and effort spent on data cleaning and standardization, with concomitant improvements in the accuracy of data. These standardized data can be uploaded to the district health information system 2 (DHIS2) One Health AMR surveillance portal, enabling the generation of reports that will help decision-makers and policy planners to combat the global problem of AMR.

## 1. Introduction

Antimicrobial resistance (AMR) is one of the top 10 global public health issues that the world is facing today and poses a serious threat to achieving the United Nations’ sustainable development goals (SDGs) related to public health, poverty, food security, and the environment [1,2]. Drug-resistant infections are linked to considerable morbidity and mortality, accounting for 700,000 deaths globally each year. Bacterial AMR-related deaths reached nearly five million globally in 2019, and it has been estimated that if sustained efforts are not made to contain AMR, these deaths could increase to 10 million annually by 2050 [3,4]. AMR is increasing day by day and is considered to be a “One Health” problem due to the interconnectedness of the human, animal, and environmental health sectors, along with the policy shortcomings in each sector. The root causes of AMR are the overuse and improper use of antibiotics in the fields of human, animal, and environmental health, as well as the spread of pathogens and resistance determinants within and between these sectors [5,6,7]. To monitor and understand this complex issue and to put effective interventions into place, a One Health surveillance strategy that integrates data about resistant organisms that are currently circulating in humans, animals, and the environment, as well as antimicrobial use in these sectors, is required [8]. Several countries adopted the Global Action Plan on AMR (GAP-AMR) after the relevant resolutions were made at the World Health Assembly in 2015 and drafted their own National Action Plans (NAPs) to tackle the growing challenge of AMR. GAP-AMR provides essential information on the emergence and occurrence of AMR for the general public, patients, healthcare providers, legislative organizations, and scientific researchers [9,10].

**Figure 1:**
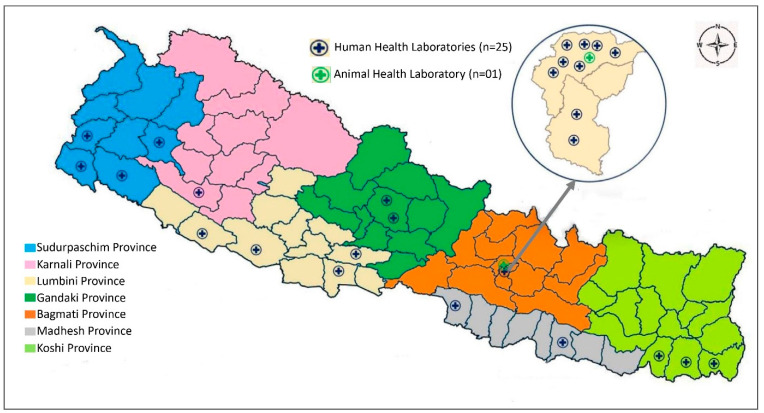
Map showing the distribution of AMR surveillance sites in the provinces of Nepal.

Nepal began its AMR surveillance program in 1999, identifying five priority bacterial pathogens (*Streptococcus pneumoniae, Haemophilus influenzae*, *Shigella* species, *Vibrio cholerae*, and *Neisseria gonorrhoeae*) and assigning nine laboratories, with the National Public Health Laboratory (NPHL) being designated as the national reference laboratory for human health and the national coordinating center for AMR surveillance in Nepal [11,12]. The national AMR surveillance program has gradually expanded; the NPHL currently has 26 participating laboratories, distributed throughout the country in all seven provinces (Figure 1) and including Nepal’s Central Veterinary Laboratory (CVL), the reference laboratory for animal health. The NPHL now monitors an additional five bacterial pathogens: *Salmonella* species (Typhi and Paratyphi), the methicillin-resistant *Staphylococcus aureus* (MRSA), *Escherichia coli*, *Klebsiella* species, and *Acinetobacter* species. All 10 are considered to be priority pathogens by Nepal’s national AMR surveillance program, which also includes the World Health Organization (WHO) global antimicrobial resistance and use surveillance system (GLASS) priority pathogens [13]. The CVL and its associated veterinary laboratories have been conducting routine and active AMR surveillance of at least four bacterial pathogens (*Escherichia coli*, *Salmonella* species, *Enterococci*, and *Campylobacter* species) in animal samples [14]. The NPHL first checks the quality of the surveillance data received from participating sentinel laboratories; it then processes, compiles, and analyzes the data and reports the surveillance findings, both nationally (to policymakers and the sentinel surveillance sites) and internationally (to WHO-GLASS, on an annual basis) [15].

The effective delivery of information generated by the AMR surveillance program requires the timely collection, processing, analysis, and reporting of AMR surveillance data [16]. However, in Nepal, the AMR surveillance data reported by sentinel laboratories to reference laboratories such as NPHL and CVL are often inconsistent, incomplete, and delayed. Thus, NPHL and CVL face many challenges in terms of cleaning, collating, analyzing, and visualizing the AMR surveillance data. This is due to erratic data reporting, arising from manual data entry into the laboratory recoding register, discrepancies in the laboratory information management systems (LIMS) used for data recording and reporting across laboratories, and constraints in terms of human resources at both the national and sentinel laboratories [17,18]. The various versions of LIMS used by different sentinel laboratories mean that the surveillance data collected arrive in different file formats, have many inconsistencies, and do not adhere to the standard reporting format. Cleaning, collating, and analyzing such data is labor-intensive and inefficient; this situation is exacerbated by the limited number of staff at the reference laboratories, who must spend a considerable amount of time and energy on this task. NPHL and CVL also face challenges in maintaining a reliable AMR surveillance data management and visualization system for evidence-based decision-making [18].

To overcome these problems regarding AMR surveillance data management in Nepal, it was necessary to develop digital tools or interfaces that were quick to implement, easy to adopt (learn, deploy, and use), and efficient, to reduce human time and effort while improving the accuracy of data management. The objective of this project was to develop a platform for One Health AMR surveillance that would enable the rapid data aggregation, visualization, and analysis of both human and animal health data. Herein, we describe our experiences in the development of reliable, convenient, efficient, and innovative digital tools for One Health AMR surveillance data management in Nepal that can be used to generate high-quality outputs and better inform policymaking to combat AMR.

## 2. AMR Surveillance Data Management Using Integrative Digital Tools

**Figure 2:**
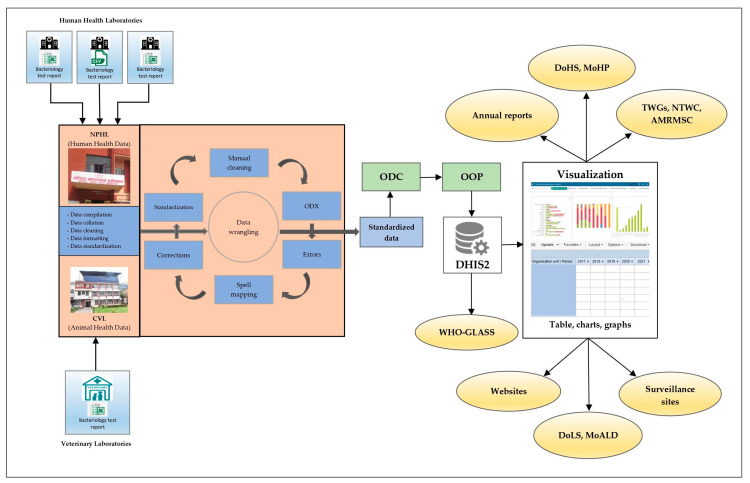
Flow diagram showing data management for the AMR surveillance program in Nepal. AMRMSC, Antimicrobial Resistance Containment Multisectoral Steering Committee; CVL, Central Veterinary Laboratory; DHIS2, district health information system 2; DoHS, Department of Health Services; DoLS, Department of Livestock Services; GLASS, global antimicrobial resistance and use surveillance system; MoALD, Ministry of Agriculture and Livestock Development; MoHP, Ministry of Health and Population; NPHL, National Public Health Laboratory; NTWC, National Technical Working Committee; ODC, open data connector tool; ODX, open data XLS transformer tool; OOP, Open Interop; TWGs, technical working groups; WHO, World Health Organization.

A situation analysis of the AMR surveillance data collection tools and methodologies in the surveillance sites, along with the data reporting, collation, cleaning, analysis, and feedback mechanisms, was conducted to identify and understand the specific issues at each stage of data management. Following this situation analysis, a series of discussions were held among experts, technical working groups, and external development partners to identify options for the best possible solutions and the potential for developing digital tools to address the aforementioned issues around data management. A desktop application (app) was developed to aid in the standardization of AMR surveillance data reported by surveillance sites. Data from the human and animal health laboratories are now processed to a standard format at the NPHL and CVL, respectively, by means of this digital tool. Once standardized, the data are uploaded and visualized in a web-based portal known as the district health information system 2 (DHIS2) [19]. A One Health AMR digital interface was developed, which enables both the NPHL and CVL to submit data and perform real-time analysis, visualization, and the reporting of AMR data when necessary (Figure 2).

### 2.1. AMR Surveillance Data Reporting Formats

The sentinel surveillance sites, which are distributed throughout Nepal, currently send human health AMR surveillance data to the NPHL via email. Similarly, the veterinary laboratories in the regions periodically submit data to CVL, either in Microsoft Excel or Word formats, via email. The AMR surveillance protocol requires that the sentinel surveillance sites share their data with the NPHL on a monthly basis; however, some sentinel sites send their data at irregular intervals. The datasheets that the NPHL currently receives are available in a variety of file formats, including comma-separated values (CSV), Excel, and WHONET files, with Excel files being the most common format and preferred by most of the sentinel sites. Each sentinel site extracts the dataset from its LIMS and sends it to the NPHL, regardless of the standard format that the NPHL recommends for reporting [15]. As the various sentinel surveillance sites use different LIMS, the data sheets that they provide do not contain information that is consistent with the standard reporting format (Tables S1–S3 in the Supplementary Materials File S1).

### 2.2. Converting and Combining Data

The first step after receiving surveillance data from a sentinel site is to convert the file into a common format, i.e., Excel. This process requires some time and effort and entails addressing the issues of any missing data, duplicate data, mandatory fields, and fractional data, i.e., data received at various frequencies and in multiple files, such as monthly data files, specimen-specific data files, etc. Any fractional data are manually combined into a single Excel file.

### 2.3. Data Cleaning and Standardization: The Open Data XLS (ODX) Transformer Tool

To overcome the data inconsistencies outlined above, the NPHL and CVL, with support from FIND (Geneva) and other technology partners, developed an innovative, bespoke tool called the open data XLS (ODX) transformer (Figure S1 in the Supplementary Materials File S2), which is used to perform the cleaning and standardization of surveillance data. ODX is a standalone tool for handling data inconsistencies, which has been designed specifically to process data from the laboratories ahead of data transmission into the One Health System. This tool has been installed at the NPHL and CVL to handle the data gathered from surveillance sites throughout Nepal. ODX solves most of the known data consistency issues, such as the misspelling of specimens, organisms, and antimicrobials, as well as addressing data completeness issues. The tool automatically makes “corrections” and identifies data quality “errors” in the spreadsheets sent by sentinel surveillance sites, based on the configurations and mapping set by the NPHL and CVL. Corrections are mapped into ODX with the help of “Spell Maps”, which is a data dictionary for AMR surveillance that stores key value pairs of data for specimens, organisms, antimicrobials, etc. (Figure S2 in the Supplementary Materials File S2). As these fields are the most prone to misspelling, Spell Maps attempts to address and convert the various spellings into a single standard form.

### 2.4. Data Upload to the DHIS2 One Health AMR Portal

After fully standardizing and cleaning a datasheet with ODX and ensuring that no errors remain unaddressed, each individual datasheet is uploaded to the DHIS2 One Health AMR portal using another tool, referred to as the open data connector (ODC) tool.

#### 2.4.1. Open Data Connector (ODC) Tool

The ODC is another dependency tool for data management in DHIS2, which helps to upload Excel spreadsheets to various servers, depending on the need. We have currently provisioned three server instances, which are further categorized as Human Health and Animal Health (Figure S3 in the Supplementary Materials File S2). The three servers comprise the following:

- Development server (Dev Server): The Dev Server is the most active instance of the server, where data uploads are performed for testing and review purposes.
- Production server (Prod Server): The Prod Server is specifically designed for the final reports and visualization after the successful reconciliation of data in the Dev Server.
- Training Server: The third server has been established for training purposes.

#### 2.4.2. Open Interop: Checking the Transmission Status of Data

The status of data transmission from the ODC to DHIS2 can be viewed using the interoperability middleware, “Open Interop”. Open Interop provides a summary of the number of transmissions received over a given period (e.g., in the past 24 h or in the past week), the server location where the data were uploaded (Dev, Prod, or Training), and the number of failed transmissions in the past 24 h and in the past 30 days. It can also provide more detailed information about the individual transmissions, including data on the requesting body and the response (Figure S4 in the Supplementary Materials File S2). The interoperability middleware helps in aggregating data from the animal and human health sectors into a common One Health data model that is implemented on DHIS2.

### 2.5. AMR Data Visualization and Dashboards

The data visualization process and dashboards are created on the DHIS2 platform once the data reconciliation process has been completed. DHIS2 is an open-source, web-based information system for data management and analysis, particularly for health information systems; it has been used to report health-related data in Nepal for many years [19]. One of the key features of DHIS2 is its ability to provide data visualization through a dashboard. We have developed several dashboards using the “Event Visualizer” and “Data Visualizer” apps available in DHIS 2.

#### 2.5.1. Dashboard Planning

A dashboard is a web page that provides an interactive way to view data in real-time. Before building a dashboard, its features are selected depending on the purpose and target audience. This will help to determine what data elements and indicators are to be included in the dashboard. Thus, prior to building any reports or dashboards, discussions are needed to determine what types of reports are needed for a given dashboard. In our case, several meetings were held between the NPHL and CVL to plan the dashboard, considering reports and other publications produced by the NPHL, CVL, WHO, and other relevant stakeholders working on AMR.

#### 2.5.2. Generating Reports

Based on the needs identified during dashboard planning, the required data variables to be reported were identified and created using the “Maintenance App” in DHIS2. Afterward, the tables, reports, charts, and visualizations were created, along with the appropriate filters. Filters allow users to customize their visualization by selecting specific data elements, indicators, time periods, and geographic regions.

#### 2.5.3. Building a Dashboard

Building a dashboard requires its layout to be configured. The DHIS2 platform allows users the flexibility to customize any dashboard by simply dragging and dropping components (reports). Once the reports have been generated, tables, charts, and other visualizations can be arranged to create a dashboard layout that conveys meaningful information to the target audience.

#### 2.5.4. Reviewing and Refining the Dashboard

Following the development of appropriate dashboards, further discussions can be carried out among expert team members to review, validate, and refine the dashboards as needed. This may involve slight variations in the data elements, indicators, visualizations, etc. Once the overall components have been finalized, the dashboards can be published or shared as required.

### 2.6. One Webpage for All Tools

As there are several different tools and IT infrastructures that must be used in the One Health AMR Surveillance System, we have created a homepage (np.amr.health; accessed on 2 April 2023) in the public domain, comprising a list of the tools used. This one-stop webpage makes it easier for users to access the specific tools that they need [20].

## 3. Outcomes and Discussion

A comprehensive representation of the surveillance data generated in various locations across the country is necessary to share information about AMR with policymakers and healthcare institutions. This information can then be used to support the development of evidence-based regulations and standards. Reliable and timely data are needed to convince policymakers to invest in long-term solutions to tackle AMR [21,22]. To achieve this, it is crucial to ensure integration and interoperability between all national data sources [1]. However, due to the discrepancies in the recording and reporting of AMR surveillance data among the sentinel laboratories, there was a pressing need for a common platform across the One Health sectors in Nepal. Therefore, to address these issues, we created a customized set of digital tools for the standardization and visualization of data in the DHIS2 portal.

In 2017, the WHO Advisory Group on Integrated Surveillance of Antimicrobial Resistance (AGISAR) published guidelines for applying a One Health approach to AMR surveillance [23]. An analysis in 2017 by Rattanaumpawan and colleagues identified a limited number of systems available for monitoring AMR in humans [24]. WHONET is a widely used LIMS software application that can manage microbiology data and track antimicrobial susceptibility. This free software is currently used by more than 130 countries. However, WHONET has several limitations in relation to capturing the microbiology data generated by hospitals and carries a high possibility of duplicate data entry. Furthermore, WHONET is unable to integrate data from the animal and environmental health sectors, although an adaptation for animal health data is in progress [24,25]. The Indian Council of Medical Research (ICMR) has designed and developed a set of three tools to improve antimicrobial resistance surveillance in India. The ICMR’s antimicrobial resistance surveillance system (i-AMRSS) is an open-source, innovative web-based application for collecting, maintaining, and analyzing AMR data from tertiary care institutions across the country. The ICMR’s Data Import App (i-DIA) is a login-based, browser-independent web API that streamlines AMR data interoperability by importing most of the LIMS data from CSV files into a centralized, one-stop data repository. The ICMR’s antimicrobial resistance surveillance system using integrative technologies (i-AMRIT) is a platform-independent, API-enabled standalone system that collects data from all labs across the country and sends lab-specific cumulative data to a single data repository. The entire system is currently being utilized to collect only human susceptibility testing data; however, it can be extended for AMR surveillance by utilizing the ‘One Health’ approach [1,26,27].

The data cleaning processes at the NPHL and CVL have been greatly improved through the use of the digital tools we developed. The ODX tool can process large datasheets in a short time, reducing the number of repetitive tasks that staff must perform, which in turn reduces the staff time and effort spent on data cleaning. It has also increased the quality of the information, as ODX maintains the accuracy of data. In addition to making automatic corrections, ODX flags errors that require manual attention. As part of the standard format of the output spreadsheet, ODX indicates any rows that have errors and any rows where corrections have been made (Table S4 in the Supplementary Materials File S1). A further useful feature of ODX is that it breaks down comma-separated values of “antibiotics”, submitted in separate “resistant”, “sensitive”, and “intermediate” columns; it then creates a new column for each antibiotic with a specific antibiotic susceptibility test (AST) result and incorporates the values “R” for “resistant”, “S” for “sensitive”, and “I” for “intermediate” in the respective antibiotics columns (Table S5 in the Supplementary Materials File S1). The ODX tool has greatly mitigated this burden, which previously required a considerable amount of time and manual effort. A key advantage of this tool is that it helps to standardize the surveillance data sent by different LIMSs, minimizes the manual data cleaning required, and addresses common dataset inconsistencies and spelling variations without any recurring cost implications. Thus, based on this cleaning process, NPHL can develop actionable steps and provide instructions, training, and monitoring to specific reporting sites to improve their existing system and solve any issues at the source. For uploading data, the system provides different environments through virtualization, which are to be used as per the priority and need. The Training, Dev, and Prod servers have been set up to allow concurrent training, development, and final access to the surveillance portal.

In addition to efficient and streamlined data cleaning, standardization, and uploading processes, the One Health AMR portal provides a single platform for the combined analysis of human and animal health data. With the power of DHIS2, it is intended to improve the overall reporting process. The DHIS2 platform allows users to display data in various formats, such as tables, charts, graphs, maps, and pivot tables [28]. It also enables users to visualize their data in a way that helps them to understand and communicate their findings to stakeholders, policymakers, and institutions. These data can then be visualized through a dashboard [19]. A dashboard can be created, based on the need for reports, charts, graphs, etc. that require a set of data elements and indicators that is determined by an AMR surveillance program. A user must apply the appropriate filters to the metadata by selecting specific data elements, indicators, time periods, etc., to customize the visualization they require. Once the required reports, tables, charts, etc. have been generated, they can be arranged to create a dashboard that can then be published or shared (Figure S5 in the Supplementary Materials File S2).

Prior to the implementation of the newer system, it took approximately 10 days, on average, to complete the manual cleaning, collating, standardizing, and visualizing the reported AMR data of one surveillance site in an Excel spreadsheet; however, this time has been significantly reduced with newly developed tools, meaning that the same tasks take less than two days on average for one surveillance site. Comparing the new system to the prior manual procedure, this newer system is able to reduce the effort required of the data analyst by more than 80%. The rate of data transmission through ODC into the DHIS2 portal is around two seconds per row. Using DHIS2, reports can be generated instantly on dynamic dashboards. When new datasheets are uploaded, the reports and dashboards are immediately updated, enabling real-time data visualization, which was not possible with the earlier manual analysis. After reviewing the reports in DHIS2, some of the reports have also been selected for publishing on the NPHL website [29] to support stakeholders with AMR-related policymaking, as well as to help clinicians to make decisions regarding antibiotic prescriptions. The reports generated from DHIS2 can also be included in AMR surveillance annual reports and newsletters. Previously, decision-makers had to rely on data analysts to perform AMR data analysis. With the current DHIS2-based system, they can access updated data analysis (reports) and dashboards, as well as evaluate trends at their convenience. Clinicians and policymakers are utilizing these AMR reports to make evidence-based decisions regarding antibiotic prescription, supportive supervision, resource allocation, and priority-setting.

Although the adoption of this newer system has not altered the entire business process of AMR data management in Nepal, it has improved the data cleaning, standardization, and visualization processes, thus improving data quality and management; it has changed the control of data access, storing data in one portal and creating real-time dynamic dashboards. All these improvements have had a significant impact on data management in AMR surveillance. The data had previously been stored on a desktop computer without a proper data warehouse, posing a security risk. Additionally, previously scattered AMR data is now streamlined and stored locally on a secured server in a data warehouse that is managed by the government. The integrated health information management system (IHIMS) in Nepal also manages health service information (including both aggregate and patient-based data) from the community level through the DHIS2 portal. The IHIMS department is rigorously training healthcare workers throughout Nepal to enable them to correctly enter their data via DHIS2. Thus, the country now has real-time patient data, entered by many healthcare facilities, which can be analyzed to inform rational, evidence-based decision-making [30]. Furthermore, there is a separate module in the system that can extract data for selected timeframes, available in Excel/CSV format, that can be used to generate reports for WHO-GLASS; this module is currently in progress and under review. There are not many tools or applications designed specifically for One Health AMR surveillance, but these fully integrated and customized tools are probably novel ones that can be used for the management of One Health AMR surveillance data.

## 4. Data Security and Confidentiality

Data security and confidentiality are essential components of any health information system. During AMR surveillance, sensitive data, such as patient medical records, diagnoses, and test results, are recorded and stored electronically. To maintain patient rights, privacy, and confidentiality, these data must be protected from unauthorized access, theft, and misuse. To ensure data security and confidentiality, primary and backup servers have been set up. In addition to this safe and secure deployment, the One Health AMR surveillance system has implemented an array of strategies and protocols for data security and confidentiality. These include data encryption, which encrypts sensitive information during transmission and storage to prevent its unauthorized access or interception, and user authentication, which implements secure user authentication protocols to ensure that sensitive data are only accessible to authorized users. AMR surveillance data governance involves managing the availability, usability, integrity, and security of data. The NPHL and CVL are committed to maintaining effective AMR data governance by ensuring that their data are consistent, trustworthy, are not misused, and are secure. Roles and access management for the AMR surveillance data and information have been developed to define each user’s access to these resources. To ensure the quality and consistency of the AMR surveillance data, data quality features are inbuilt within the system.

## 5. Conclusions and Recommendations

This article describes how the use of digital tools in Nepal has contributed to an effective and efficient AMR surveillance program, one that is built on quality data, informative analysis, and clear presentation. These tools are currently being deployed at the NPHL and CVL for data management in the human and animal health sectors. These tools are more innovative than those in earlier systems and greatly assist professionals involved in AMR surveillance data management by reducing time and labor costs. Currently, data from both sectors are being uploaded into the DHIS2 common interface, known as “One Health” AMR surveillance, and the findings thus generated can be used by stakeholders, including human and animal healthcare institutions, for improved AMR-related decision-making and policy generation. These tools can also be used on-site by sentinel surveillance locations to standardize their data and upload them directly to DHIS2, once they are provided with appropriate training for staff and suitable data management systems. In the future, customized versions of these tools could be used to handle AMR surveillance data in other sectors, such as the environment.

## Data Availability

Not applicable.

## Supplementary Materials

The following supporting information can be downloaded at: https://www.mdpi.com/article/10.3390/tropicalmed8060291/s1, File S1: Table S1: Standard format for reporting AMR surveillance data to the NPHL; Table S2: Sample format of the datasheet submitted by sentinel surveillance site A to NPHL; Table S3: Sample format of the datasheet submitted by sentinel surveillance site B to the NPHL; Table S4: A summary of the errors identified and corrections made by ODX in the formatted sheet; Table S5: Breakdown via ODX of the comma-separated values of “antibiotics” into separate columns with antimicrobial susceptibility testing results. File S2: Figure S1: The open data XLS (ODX) transformer tool; Figure S2: Spell Maps used to convert spelling variations into a common form; Figure S3: The open data connector (ODC) tool for uploading datasheets to the DHIS2 platform; Figure S4: Data transmission details in Open Interop; Figure S5: The data visualization tools and dashboard in the DHIS2 portal.
