# Supplementary material for: Integrative Digital Tools to Strengthen Data Management for Antimicrobial Resistance Surveillance in the “One Health” Domain in Nepal
Source: Trop Med Infect Dis. 2023 May 25;8(6):291. doi: 10.3390/tropicalmed8060291 (PMC10300725; doi:10.3390/tropicalmed8060291)
Supplement: Supplementary file 1 [file tropicalmed-08-00291-s001.zip › File S1.pdf]

Table S1. Standard format for reporting AMR surveillance data to the NPHL [15].

Patient Id/No	Name	Age	Gender	OPD/IPD	Date of admission	Institution	Department	Location	Specimen	Specimen collection date	Organism	Antibiotics						
												Ab1	Ab2	Ab3	Ab4	Ab5	Ab6	Ab7

Ab; antibiotics tested against the organism.

Table S2. Sample format of the datasheet submitted by sentinel surveillance site A to the NPHL.

SN	Name	Sample	Patient id	Age	Sex	Sample taken Date	Finding	Sensitivity	Intermediate	Resistant
1	AA	Urine	111	20	MALE	2022-01-01	Culture sterile after 24 hrs of aerobic incubation at 37°C			
2	BB	Blood	222	25	FEMALE	2022-01-01	Culture sterile after 24 hrs of aerobic incubation at 37°C			
3	CC	Urine	333	45	FEMALE	2022-01-01	Escherichia coli	Ceftriaxone, Nitrofurantoin, Piperacillin, Tigecycline	Levofloxacin	Co-Trimoxazole, Gentamicin, Ofloxacin
4	DD	Sputum	444	60	FEMALE	2022-01-01	Enterococcus faecalis	Levofloxacin, Linezolid, Nitrofurantoin, Teicoplanin, Vancomycin		
5	EE	CSF	555	70	FEMALE	2022-01-02	Culture sterile after 24 hrs of aerobic incubation at 37°C			

Table S3. Sample format of the datasheet submitted by sentinel surveillance site B to the NPHL.

Lab No	Age	Gender	Origin	Test Name	TestDate	Bacteria	Amikacin	Amoxycillin	Amoxycillin /Clavulanic Ac	Ampicillin	Cefepime	Cefixime	Cefotaxime	Ceftazidime
1	038	M	OPD	Urine C/s	1/1/2021	Escherichia coli	S	R	R		R	R	R	R
2	017	F	OPD	Urine C/s	1/1/2021	No Growth								
3	064	F	OPD	Urine C/s	1/1/2022	Escherichia coli		S				S	S	
4	030	M	OPD	Blood C/S	1/1/2022	No Growth								
5	040	F	OPD	CSF C/S	1/1/2022	No Growth								

Table S4. A summary of the errors identified, and corrections made by ODX in the formatted sheet.

Error	Error description	Correction	Correction description	Sitetype	Ast_id	Spreadsheet_name
		True	Gender changed from F to "f", Bacteria changed from No growth to "No growth",	NPHL	186f460be110.62bec424d8cec	NPHL_April_2022.xlsx
True	Sample is not recognised,	True	Gender changed from M to "m", Bacteria changed from No growth to "No growth",	NPHL	186f460be110.bbf68e8924c0f	NPHL_April_2022.xlsx
True	Bacteria is not recognised,	True	Gender changed from M to "m",	NPHL	186f460be120.4f8db92d8b3e1	NPHL_April_2022.xlsx
		True	Gender changed from F to "f", Bacteria changed from No growth to "No growth",	NPHL	186f460be140.7ffc98fbe8335	NPHL_April_2022.xlsx
True	Bacteria is not recognised,	True	Gender changed from M to "m",	NPHL	186f460be140.b7559f3edcbc7	NPHL_April_2022.xlsx
		True	Gender changed from M to "m", Bacteria changed from No growth to "No growth",	NPHL	186f460be4b0.212376b11d7d	NPHL_April_2022.xlsx
True	Bacteria is not recognised,	True	Gender changed from F to "f",	NPHL	186f460be4b0.764785f737688	NPHL_April_2022.xlsx
True	Sample is not recognised,	True	Gender changed from M to "m", Bacteria changed from No growth to "No growth",	NPHL	186f460be4b0.243c58e6b1001	NPHL_April_2022.xlsx

Note: During data cleansing by ODX, the user is provided with a summary of what has been auto-corrected based on the configurations and mapping and where errors have been found. An "error" column in the formatted sheet states 'true' if an error identified, and the adjacent column with a detailed "error description". A "correction" column states 'true' if an automatic correction has been done, and the adjacent column with a detailed correction description made by ODX.

Table S5. Break down via ODX of the comma-separated values of "antibiotics" into separate columns with antimicrobial susceptibility testing results.

S.N.	Name	Age	Sex	TestDate	Specimen	Result	Resistant	Intermediate	Sensitive
1	AA	12	M	2022/01/02	Sputum	Klebsiella species	Ciprofloxacin	Levofloxacin	Gentamicin, Amikacin, Cefotaxime, Meropenem
2	BB	15	F	2022/01/02	Urine	E. coli	Ampicillin, Cefotaxime	Ciprofloxacin	Gentamicin, Amikacin, Meropenem, Levofloxacin
3	CC	24	F	2022/01/02	Blood	No growth			
4	DD	30	M	2022/01/03	Urine	E. coli	Ciprofloxacin, Levofloxacin	Gentamicin	Ampicillin, Cefotaxime, Amikacin, Meropenem

After ODX Cleansing

S.N.	Name	Age	Sex	TestDate	Specimen	Result	Ampicillin	Ciprofloxacin	Levofloxacin	Gentamicin	Amikacin	Cefotaxime	Meropenem
1	AA	12	M	2022/01/02	Sputum	Klebsiella species		R	I	S	S	S	S
2	BB	15	F	2022/01/02	Urine	E. coli	R	I	S	S	S	R	S
3	CC	24	F	2022/01/02	Blood	No growth							
4	DD	30	M	2022/01/03	Urine	E. coli	S	R	R	I	S	S	S

Note: In addition to spelling errors and automatic correction, a further useful feature of ODX is that it breaks down comma separated values of "antibiotics", submitted in separate "resistant", "sensitive", and "intermediate" columns, and creates a new column for each antibiotic with a specific antibiotic susceptibility test (AST) result and incorporates the values "R" for "resistant", "S" for "sensitive", and "I" for "intermediate" in the respective antibiotics columns.

.
