# Supplementary material for: Integrative Digital Tools to Strengthen Data Management for Antimicrobial Resistance Surveillance in the “One Health” Domain in Nepal
Source: Trop Med Infect Dis. 2023 May 25;8(6):291. doi: 10.3390/tropicalmed8060291 (PMC10300725; doi:10.3390/tropicalmed8060291)
Supplement: Supplementary file 1 [file tropicalmed-08-00291-s001.zip › File S2.pdf]

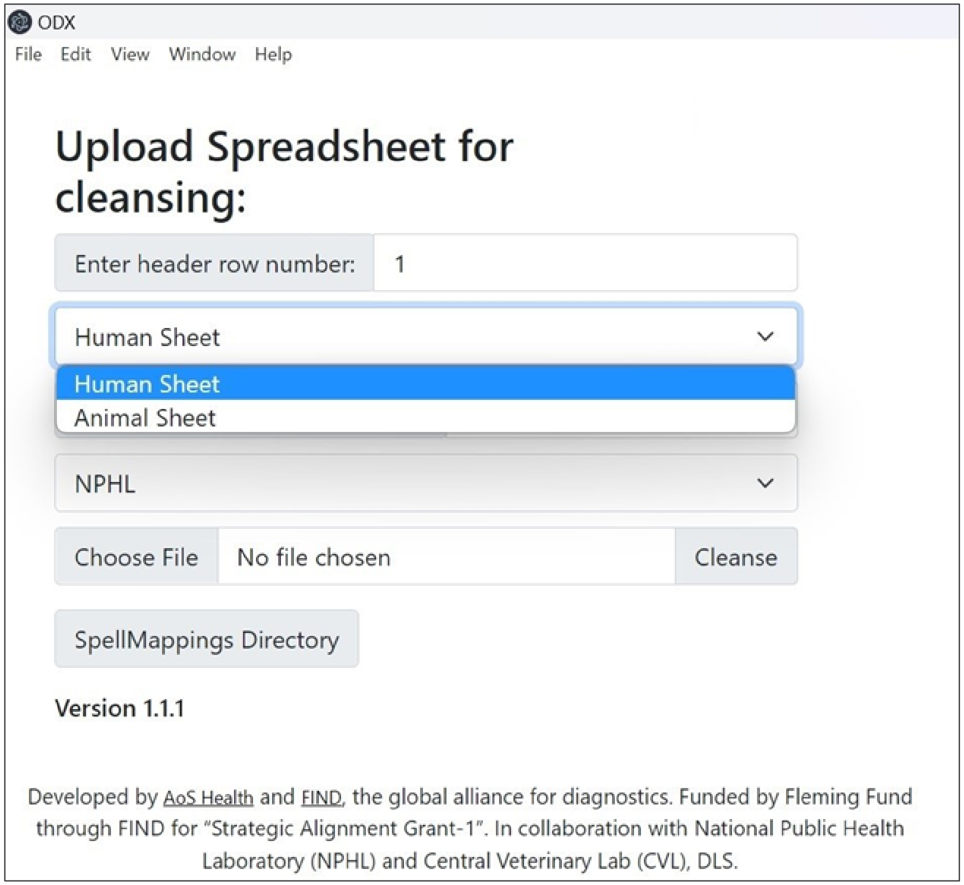

Note: The figure shows the User Interface of ODX tool, where user can load excel file and start standardizing process. There are four fields to be inputted by user. The first field is for the header row number of excel sheet; second one is for selecting Human or animal health data; third is for laboratory code and fourth one is for browsing the excel file.

Figure S1. The open data XLS (ODX) transformer tool.

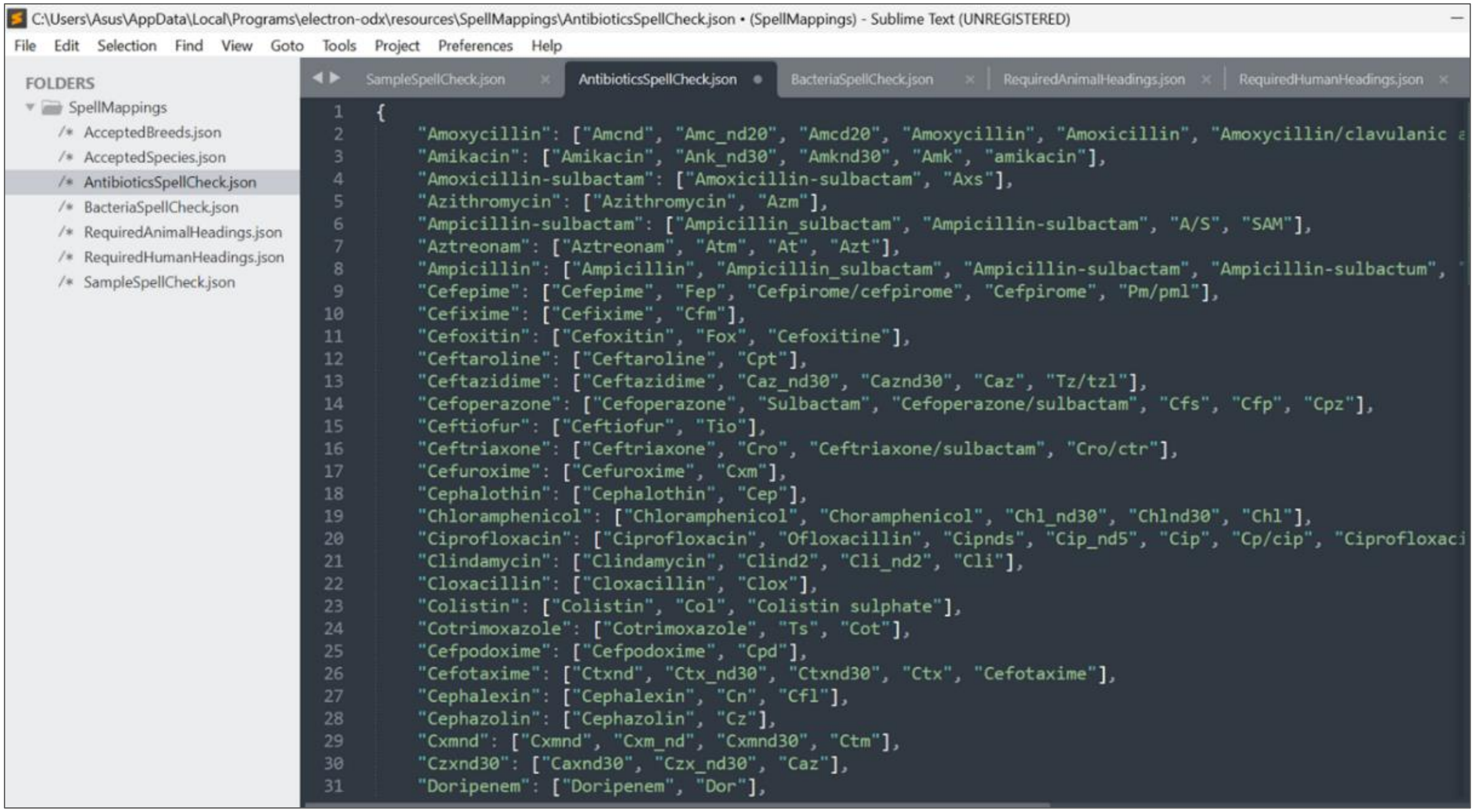

Note: The ODX tool is dependent upon Spell mappings for Antibiotics, Specimen, Bacteria, Breed and required headings. The figure depicts the range of spellings (on the right) are being mapped to a standard spelling (on the left) as key value pairs.

Figure S2. Spell Maps used to convert spelling variations into a common form.

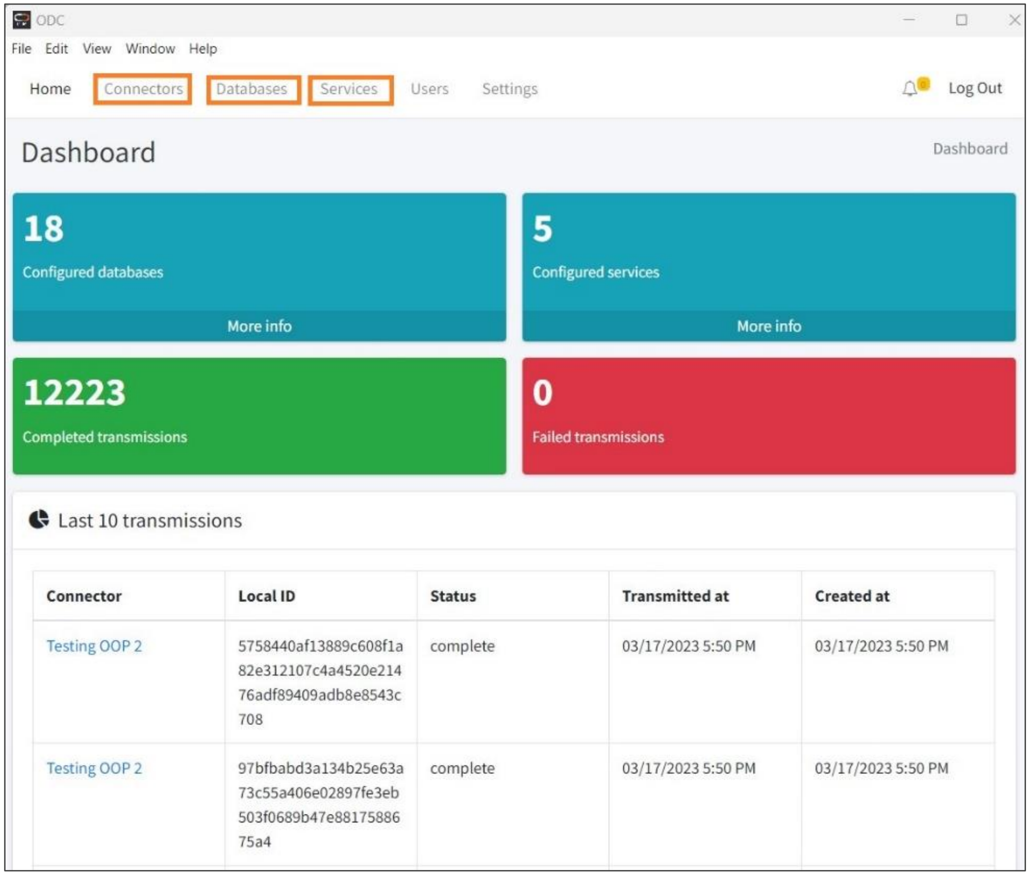

Note: The figure shows ODC tool that is used to upload data into DHIS2 portal. The three main menu highlighted here have the following functionalities. ‘Services’ has the list of URLs for instances of server. Databases is used to point to excel sheet formatted by ODX. The connectors is used to create connection between database and service while allowing data to sync.

Figure S3. The open data connector (ODC) tool for uploading datasheets to the DHIS2 platform.

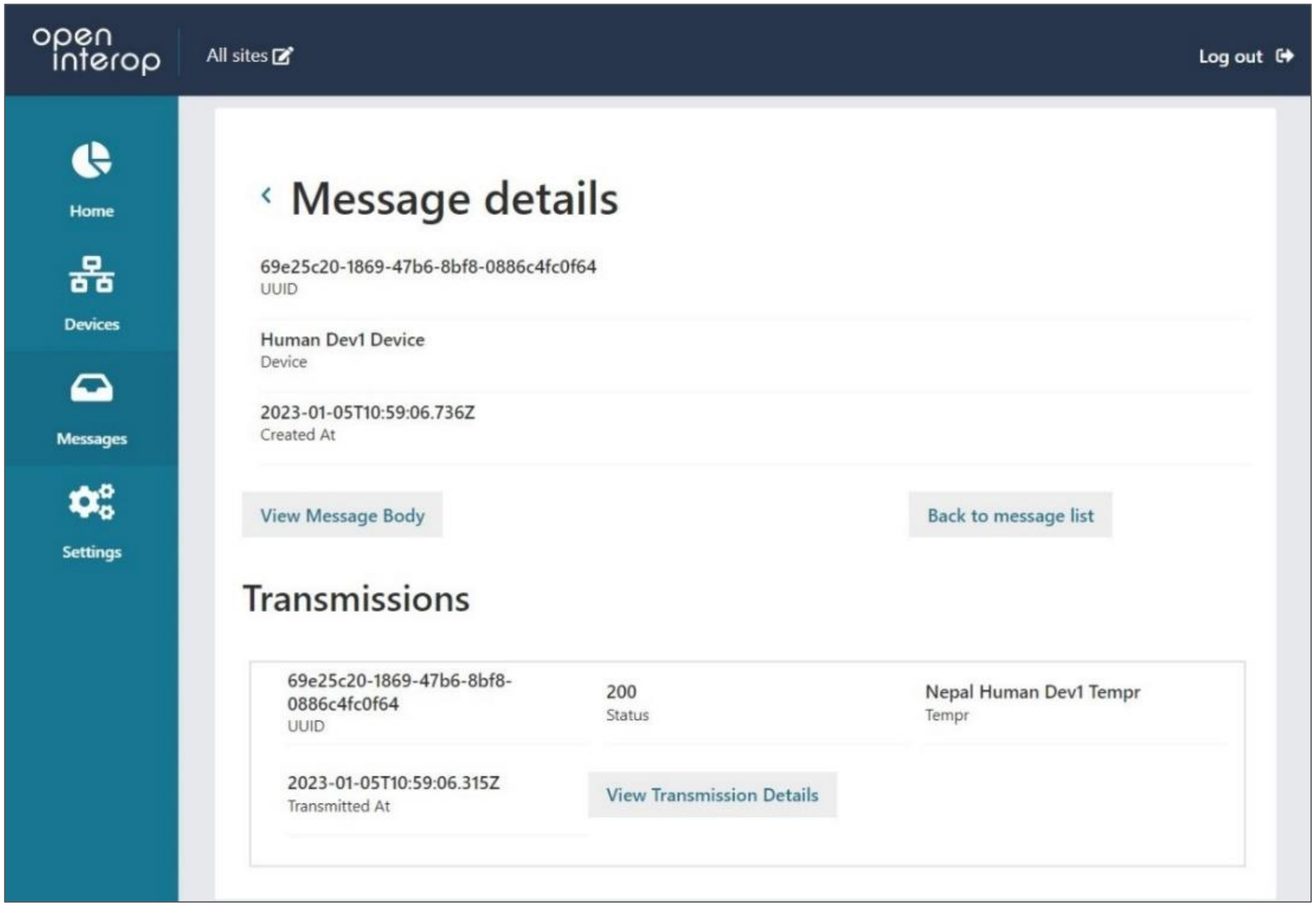

Note: The figure shows one of the message in Open Interop. The status 200 shows that the transmission has been successful. In case of troubleshooting any data not transmitted, user can refer to 'View Transmission Details' to see the reason.

Figure S4: Data transmission details in Open Interop.

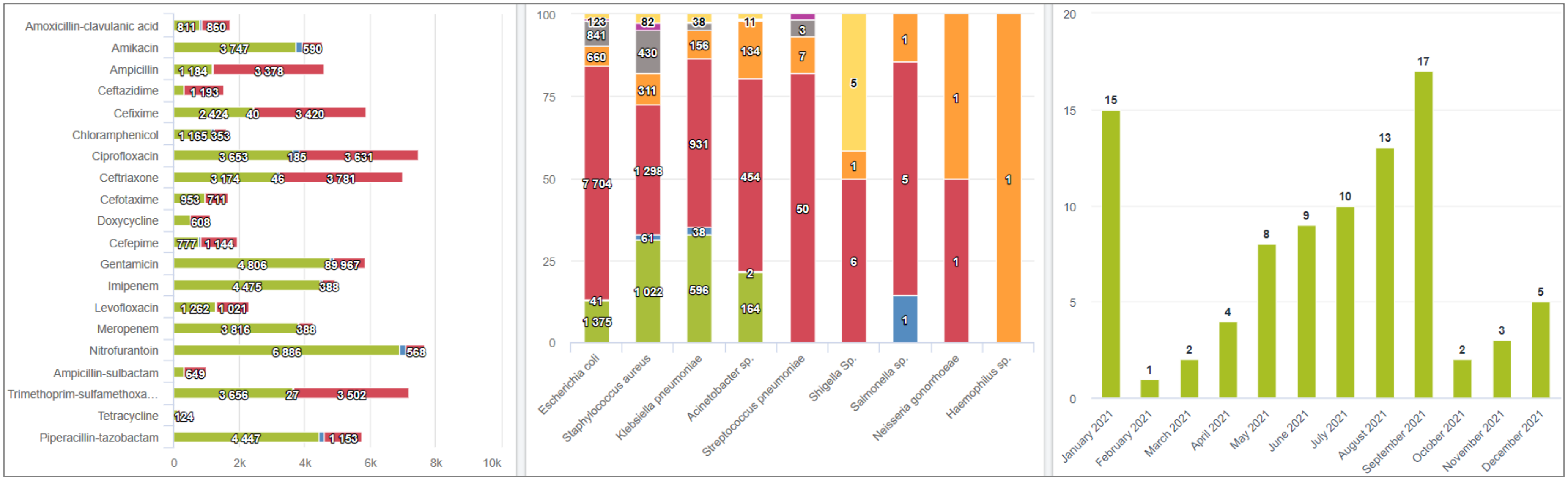

Note: The figure shows some of the report formats in DHIS2 dashboard. The exact details of data variables are not shown here, and it only presents how the data visualization appears in DHIS2 dashboard.

Figure S5. The Data visualization tools and dashboard in the DHIS2 portal.
